# Electroactive electrospun nanoplatform combined with electrical stimulation modulates anti-inflammatory macrophage polarization for enhanced wound healing

**DOI:** 10.3389/fbioe.2025.1739989

**Published:** 2026-01-08

**Authors:** Yajun Zhang, Ye Tang, Baicheng Lu, Fang Li, Kai Yu

**Affiliations:** 1 Trauma Treatment Center, Emergency Department, Beijing Genertec Aerospace Hospital, Beijing, China; 2 Key Laboratory of Trauma and Neural Regeneration, Ministry of Education, Peking University, Beijing, China; 3 National Center for Trauma Medicine, Peking University People’s Hospital, Beijing, China; 4 Trauma Medicine Center, Peking University People’s Hospital, Beijing, China; 5 Department of Orthopedics, Beijing Genertec Aerospace Hospital, Beijing, China

**Keywords:** anti-inflammatory, CNT, electrical stimulation, immune regulation, macrophage polarization, tissue regeneration

## Abstract

**Introduction:**

Immune regulation is critical for tissue repair, particularly through the polarization of anti-inflammatory macrophages. While biological and chemical stimuli can modulate macrophage polarization, the effects of physical stimuli remain underexplored. This study investigates the use of an electroactive nanofibrous scaffold combined with exogenous electrical stimulation (ES) to modulate macrophage polarization for tissue regeneration.

**Methods:**

An electroactive, aligned nanofibrous scaffold composed of polyurethane and carbon nanotubes (PU/CNT) was fabricated via electrospinning. Its ability to modulate macrophage polarization was assessed in vitro and in vivo under exogenous ES. Evaluations included biocompatibility tests, analysis of macrophage phenotype-specific gene (Arg1, IL-10, TNF-α, IL-6) and protein (IL-10) expression via qPCR, ELISA, and immunohistochemistry, and in vivo wound healing assessment.

**Results:**

The nanofibrous scaffold exhibited excellent conductivity and good biocompatibility both in vitro and in vivo. Exogenous ES significantly promoted the polarization of macrophages toward the anti-inflammatory M2 phenotype. This was confirmed by the upregulation of M2-associated genes (Arg1, IL-10) and the protein IL-10, alongside the downregulation of M1-associated genes (TNF-α, IL-6). *In vivo* histological analysis demonstrated that ES can significantly accelerate wound healing.

**Discussion:**

This work establishes that the conductive PU/CNT scaffold can effectively deliver exogenous ES to polarize macrophages toward a regenerative phenotype. It provides a novel strategy for immune modulation and a promising tool for advancing macrophage-based therapies in tissue engineering.

## 1 Introduction

Tissue injury has emerged as one of the major global health challenges, with more than 14 million people suffering from it annually (Martinengo et al., 2019). The repair of tissue represents one of the most intricate biological processes in human life. To restore tissue integrity and homeostasis following an injury, various intracellular and intercellular pathways must be activated and coordinated instantaneously (Gurtner et al., 2008). Across all organ systems, the body’s response to injury can be categorized into four distinct phases, including clot formation, inflammation, new tissue formation, and tissue remodeling (Werner and Grose, 2003). Within this process, the immune microenvironment plays a crucial role in tissue repair. Numerous studies have demonstrated that modulating the immune microenvironment can promote the repair of tissues such as bone (Fu et al., 2025; Yang et al., 2023), nerve (Liu et al., 2024; Fan L. et al., 2022), and skin (Valvis et al., 2015; Yang et al., 2025).

Macrophages, as a crucial component of the immune microenvironment, possess a wide range of functions, including tissue repair and regulation of homeostasis and immunity (Zhang et al., 2024; Qi et al., 2024). Therefore, modulating macrophages serves as a significant means of controlling the immune microenvironment for tissue repair. This can be achieved by regulating their phagocytic function, migration and differentiation to influence their immune response behavior (Chen et al., 2023a; Groh et al., 2018). Furthermore, regulating the phenotypic transformation of macrophages has emerged as a research hotspot in the biomedical field in recent years. The phenotypic transformation of macrophages from pro-inflammatory (M1) to anti-inflammatory (M2) alters their cytokine secretion profile, promoting the secretion of anti-inflammatory factors such as IL-10 and Transforming Growth Factor (TGF)-β1, thus playing a profound role in immune regulation (Wang et al., 2024; Domínguez-Andrés and Netea, 2019).

Traditionally, it was believed that the functions of immune cells were entirely modulated by the numerous biochemical signals they receive from the environment (Koppes et al., 2016; Willand et al., 2016; Anand and Chou, 1992). In recent years, it has been discovered that immune cells possess the same electrical properties as other human cells. Physical signals present in the cellular microenvironment also play a crucial role in determining the activity of immune cells, among which electrical stimulation (ES) is a potential physical signal (Zhao et al., 2020; Roy and Jhunjhunwala, 2024). Studies have indicated that exogenous ES can facilitate the transformation of macrophages into an anti-inflammatory (M2) phenotype, demonstrating beneficial effects in tissue repair (Hoare et al., 2016; Li J. et al., 2021). Therefore, the combination of ES and wound dressings to promote immune modulation during the tissue repair represents a breakthrough in the design of novel wound dressings.

The design of scaffold materials with good electrical conductivity is required for ES combined with wound dressings. The main method for fabricating conductive materials involves hybridizing polymers with conductive nanomaterials (Radhakrishnan et al., 2015). Previous research has employed 3D printing technology to produce conductive scaffolds with complex structures, better simulating the tissue microenvironment for various types of tissue repair (Lee et al., 2024; Yu et al., 2021). Nowadays, electrospun aligned fibrous scaffolds have emerged as a significant scaffold preparation method due to their excellent mechanical properties and biocompatibility (Xue et al., 2019; Carvalho et al., 2019; Allison et al., 2017). Polyurethane (PU) is a thermoplastic polymer with a series of excellent properties, such as good mechanical properties, superior elastic performance, high durability, good biocompatibility and biodegradability (Zhang et al., 2023a; Ghorbani et al., 2020; Pedicini and Farris, 2003),making it a promising scaffold material in tissue repair when combined with the unique characteristics of nanofiber structures through electrospinning technology (Mi et al., 2017; Kucinska-Lipka et al., 2015).

The conductive nanomaterials used in conductive scaffolds, such as polypyrrole (PPY), polyaniline (PANI), poly(p-phenylene vinylene) (PPV), have shown great potential for biological stimulation through *in-situ* charge transfer (Dong et al., 2020). However, most of these conductive materials are non-biodegradable or have poor biodegradability, which may cause immune responses after long-term implantation. Additionally, their complex chemical structures make them difficult to process and thus limit their widespread use (Wang et al., 1999; Ghasemi-Mobarakeh et al., 2011; Chen et al., 2025). In contrast, carbon nanotubes (CNTs) stand out as widely used conductive nanomaterials due to their strong mechanical properties, conductivity, high biosafety, and relative ease of manufacture and functionalization. Currently, numerous studies have utilized CNTs to prepare conductive scaffolds for tissue repair, confirming their effectiveness and feasibility in scaffold fabrication (Zhang et al., 2023b; Parikh et al., 2023).

Therefore, in this study, we employed electrospinning technology to fabricate conductive PU/CNT fibrous scaffolds. By combining these scaffolds with exogenous ES, we cultured macrophages on the conductive scaffolds to evaluate the impact of both the conductive scaffolds and exogenous ES on macrophages. And a rat model of wound healing was established to evaluate their effects on wound repair. Our investigation aimed to explore their roles in macrophage phenotypic transformation and cytokine secretion, and to assess their functions in wound repair *in vivo*, providing valuable guidance for the future development of tissue repair biological scaffold.

## 2 Materials and methods

### 2.1 Materials

PU was purchased from Sigma-Aldrich (Shanghai, China, No.659258). Multiwalled carbon nanotubes (MWCNTs) were purchased from Beijing HongHu LianHe HuaGong ChanPin Co. Ltd. (China). N, N-dimethylformamide (DMF) was obtained from Shanghai Macklin Biochemical Technology Co., Ltd. All other organic reagents were purchased from SINOPHARM (China), and all biological reagents were purchased from Sangon Biotech (Shanghai, China). The mouse RAW264.7 macrophage cell line was purchased from Beijing Vital River Laboratory Animal Technology, Co. (Beijing, China).

### 2.2 Preparation of electrospun nanofibrous scaffolds

Nanofibrous scaffolds were fabricated by electrospinning under optimized conditions, as follows: a 7% (w/v) PU spinning solution was prepared by dissolving 0.7 g PU in 10 mL DMF and stirring at room temperature for 12 h. PU/CNT spinning solution was made by adding a specific quantity of MWCNTs (PU/CNT at 1,000:1 ratio). These two spinning solutions were electrospun at a temperature of 21 °C and relative humidity of 50%. Spinning solutions were placed into 20 mL syringes with stainless needles, and electrospun at a flow rate of 1.5 mL/h through a CNC injection pump at 20 kV voltage. The nanofibrous scaffolds were collected in an antistatic nonwoven fabric and the acceptance distance was set at 17 cm. After vacuum drying overnight, the PU and PU/CNT membranes were obtained for subsequent experiments.

### 2.3 Characterization of nanofibrous scaffolds

The morphological structure of nanofibrous scaffolds was viewed by scanning electron microscopy (SEM, HITACHI SU8010, Japan). The membranes were sputter-coated with a 7 nm layer of gold and then observed at an accelerating voltage of 10 kV. The fiber diameters of membranes were measured from SEM photographs by ImageJ software (National Institutes of Health, United States). The internal structure of the PU and PU/CNT membranes was observed through a transmission electron microscope (TEM) (H-800, Hitachi, Tokyo, Japan). The conductivity of PU and PU/CNT membranes was measured using a four-point probe system (RTS-9, 4 PROBES TECH, Guangzhou, China).

### 2.4 Cell cultivation and cellular intervention

The mouse RAW264.7 macrophage cell line was selected for its widespread use in immunomodulation studies and high sensitivity to physical and chemical stimuli, including ES and nanomaterials (Chen et al., 2023a). RAW264.7 cells were cultured in high-glucose DMEM supplemented with 10% FBS and 1% penicillin–streptomycin. Upon reaching 80% confluence, the cells were dissociated and then seeded on PU and PU/CNT membranes at a density of 5 × 10^4^ cells/cm^2^. After 24 h to allow attachment, the cells of PU/CNT group were exposed to a direct current (DC) electric field (150 mV/mm) for 10 min per day (PU/CNT + ES group). In brief, the electric field was applied through two parallel electrodes that were fixed at the opposite end of PU/CNT membrane and connected to a DC power supply (XJ17333L, Shenzhen, China). Cells cultured on PU membrane without ES (PU group) and PU/CNT membrane without ES (PU/CNT group) were used as controls. All cells were cultured in a humidified incubator with 5% CO_2_ at 37 °C. The culture medium was renewed every 2 days.

### 2.5 Cell proliferation assay

After culturing for 1, 3 and 5 days, the proliferation of RAW264.7 cells in each group were evaluated using a Cell Counting Kit-8 (CCK-8) (Dojindo, Tokyo, Japan) according to the manufacturer’s instructions. In brief, the cells were washed twice with phosphate-buffered saline solution (PBS) and incubated with fresh medium containing 10% CCK-8 solution at 37 °C. After 2 h, the absorbance at 450 nm wavelength was measured using a microplate reader (Model 680; BioRad, CA, United States).

### 2.6 Live/Dead cell staining

Live/dead cell staining was used to detect viability and proliferation of RAW264.7 cells on day 3 of three groups, respectively. Fluorescence images were captured by a confocal laser scanning microscope (CLSM, OLYMPUS FV1000, Japan). Live cells showed green fluorescence and dead cells showed red fluorescence. Cell numbers were counted with an automatic cell counter (Countstar Biotech, ALIT Life Science, China).

### 2.7 Immunofluorescence staining

Arginase-1 (Arg1) and Inducible Nitric-Oxide Synthase (iNOS) are two crucial enzymes in macrophages that serve as indicators of macrophage phenotype (Kieler et al., 2021). RAW264.7 cells cultured on nanofibrous scaffolds for 3 days were fixed with 4% (w/v) paraformaldehyde (PFA) at 4 °C overnight, rinsed three times with PBS, permeabilizated with 0.5% Triton X-100 for 15 min, and blocked with 1% bovine serum albumin (BSA) solution for 60 min. Then, the cells were incubated with rabbit polyclonal antibodies against Arg1 and iNOS proteins overnight at 4 °C respectively and washed with PBS. Secondary goat anti-rabbit IgG-Rhodamine was added, and 1 mg/mL 40,6-diamidino-2-phenylindole (DAPI) was used to stain cell nuclei. Confocal laser scanning microscopy (CLSM) was used to observe and capture fluorescence images.

### 2.8 Quantitative real-time polymerase chain reaction (PCR)

Total RNA was extracted from treated RAW264.7 cells on day 3 using TRIzol reagent (Invitrogen, United States) following the manufacturer’s protocol. Complementary DNA (cDNA) was synthesized from the isolated RNA using the ReverTra Ace qPCR RT Kit (FSQ-201; TOYOBO) according to the provided instructions. Quantitative reverse transcription PCR (RTqPCR) was performed to valiate the results of RNA sequencing by using the PrimeScript RT Master Mix (TaKaRa, Japan) and quantified with the CFX96 Real-Time PCR System (Bio-Rad). Target gene expression levels were normalized to the housekeeping gene GAPDH using the 2^−ΔΔCt^ method (Livak et al., 2001). Each experimental group included at least three independent replicates to ensure data reliability.

### 2.9 Enzyme linked immunosorbent assay (ELISA)

The cell culture medium of all groups was collected on day 3, and proinflammatory cytokine (TNF-*α*, IL-6 and IL-10) production was assessed by ELISA kits (Invitrogen) according to the manufacturer’s protocols.

### 2.10 Animals and surgical procedures

All animal experiments were approved by the Animal Ethics Committee of Peking University People’s Hospital (Approval No. 2023PHE088). A total of 40 female SD rats (six to 8 weeks) from the Beijing Vital River Laboratory Animal Technology Co., Ltd. (Beijing, China) were randomly distributed into four groups (n = 10/group): Control group, PU group, PU/CNT group and PU/CNT + ES group, each with four assessment time points: day 0, day 3, day 7 and day 14. All rats were anesthetized with 5% isoflurane (RWD Life Science, Shenzhen, China) inhalation for 5 min for induction, followed by maintenance under 3% isoflurane for subsequent procedures. After being shaved and disinfected, the skin defect wound with a diameter of 1 cm was made in the middle of the dorsal side with a skin punch. Later, the wound surfaces were covered with PU or PU/CNT, designated as the PU group and PU/CNT group, respectively. In the ES group, two parallel carbon rubber electrodes were fixed on the shaved dorsal skin of the rats, positioned to ensure the wound and the overlying PU/CNT dressing were centered between them. A daily 10-min direct current ES at an intensity of 100 mV/cm was then delivered via a DC power supply. This specific parameter set was chosen following a review of prior studies (Chen et al., 2025; Vijayavenkataraman et al., 2019; Wei et al., 2023), as it demonstrated efficacy in promoting tissue regeneration, high biological safety, and operational practicality. This group was accordingly designated the PU/CNT + ES group. The wound region of all groups was compared with Control group. In order to record the healing process of the wound, the wound was photographed at 0, 3, 7 and 14 days after treatment. The calculation formula of wound healing rate is: wound healing rate = (A_0_ - A)/(A) × 100%, where A_0_ is the initial wound area and A is the current wound area.

### 2.11 Histological and immunohistochemical analysis

Rats were euthanized using a 50% volume per minute displacement rate of carbon dioxide for 10.5 min to ensure irreversible euthanasia on day 7 and 14 (Hickman, 2022). Later, specimens were collected and fixed in 4% PFA for 24 h. The specimens were then embedded in paraffin for routine histological hematoxylin-eosin (HE) staining and Masson’s trichrome (MT) staining analysis. All histological measurements, including wound width and granulation tissue thickness, were performed at the widest point of the wound to ensure consistency. The levels of TNF-ɑ and IL-10 was detected by immunofluorescence staining. HE staining of the heart, lung, liver, spleen, and kidney from each group was examined to evaluate biosafety *in vivo*. All images were taken using fluorescence microscopy.

### 2.12 Statistical analysis

Quantitative results were obtained for at least 5 samples in each group, and the experiment was repeated for at least 3 times. All the data were presented as means ± standard deviation (SD). Statistical comparisons among groups were analyzed by one-way ANOVA with Tukey postmortem test using Stata 19.0, with p < 0.05 considered as statistically significant differences. The statistical tests conducted in this study are also represented by legends: ∗p < 0.05, ∗∗p < 0.01.

## 3 Results

### 3.1 Characterization of nanofibrous scaffolds

**FIGURE 1:**
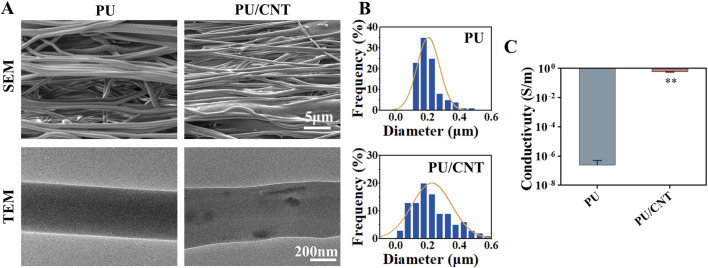
Characterization of PU and PU/CNT nanofiber membranes. **(A)** SEM and TEM images of the nanofiber membranes. **(B)** Fiber diameter distribution of the nanofiber membranes (n = 100). **(C)** Electrical conductivity of the nanofiber membranes (n = 3). Data are expressed as mean ± SD; *p < 0.05; **p < 0.01.

The PU and PU/CNT nanofibrous scaffolds were fabricated according to the aforementioned method. SEM results indicated that the electrospun fibers exhibited a continuous morphology with parallel arrangement (Figure 1A). The average fiber diameter of the PU/CNT group (0.227 ± 0.12 um) was slightly larger than that of the PU group (0.205 ± 0.07 um), but the difference was not statistically significant (Figure 1B). TEM images revealed the internal structure of both PU and PU/CNT nanofiber membranes, where the position of CNTs within the PU/CNT fibers could be clearly identified (Figure 1A). Electrical conductivity measurements for PU and PU/CNT membranes were 10^–6^ and 10^0^ S/m, respectively, demonstrating a statistically significant increase in conductivity of the fiber membrane upon CNT addition (**p < 0.01) (Figure 1C).

### 3.2 Cytocompatibility of nanofibrous scaffolds

**FIGURE 2:**
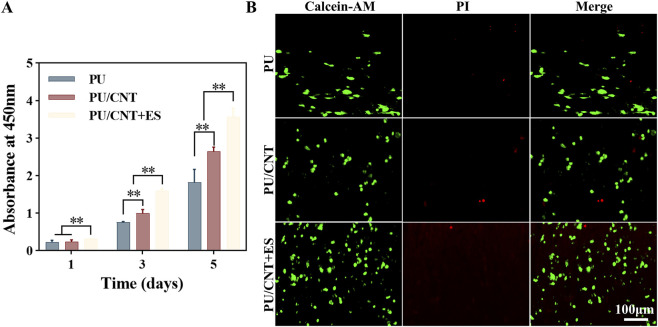
Biocompatibility of electrospun fibrous membranes with ES. **(A)** Results of CCK-8 for RAW264.7 cells on days 1, 3, and 5 (n = 3). **(B)** Results of Live/dead staining for RAW264.7 cells on day 3 (n = 3). Data are expressed as mean ± SD; *p < 0.05; **p < 0.01.

The biocompatibility of nanofiber membranes and exogenous ES was investigated through *in vitro* cultivation of RAW264.7 macrophages. Results from the CCK-8 determination revealed that RAW264.7 cells continuously proliferated on days 1, 3, and 5 in all three groups. Notably, the cell proliferation level in PU/CNT + ES group was significantly higher than that in PU group and PU/CNT group (Figure 2A). Meanwhile, in live/dead staining results, all groups exhibited good cell viability on day 3, showing strong green fluorescence. PU/CNT + ES group displayed the densest green fluorescence, while red fluorescence was rarely observed in all groups (Figure 2B). These findings suggest minimal cytotoxicity of the nanofiber membranes and exogenous ES, indicating no significant impact on the viability of RAW264.7 cells.

### 3.3 Effect of ES on the behavior of macrophages

**FIGURE 3:**
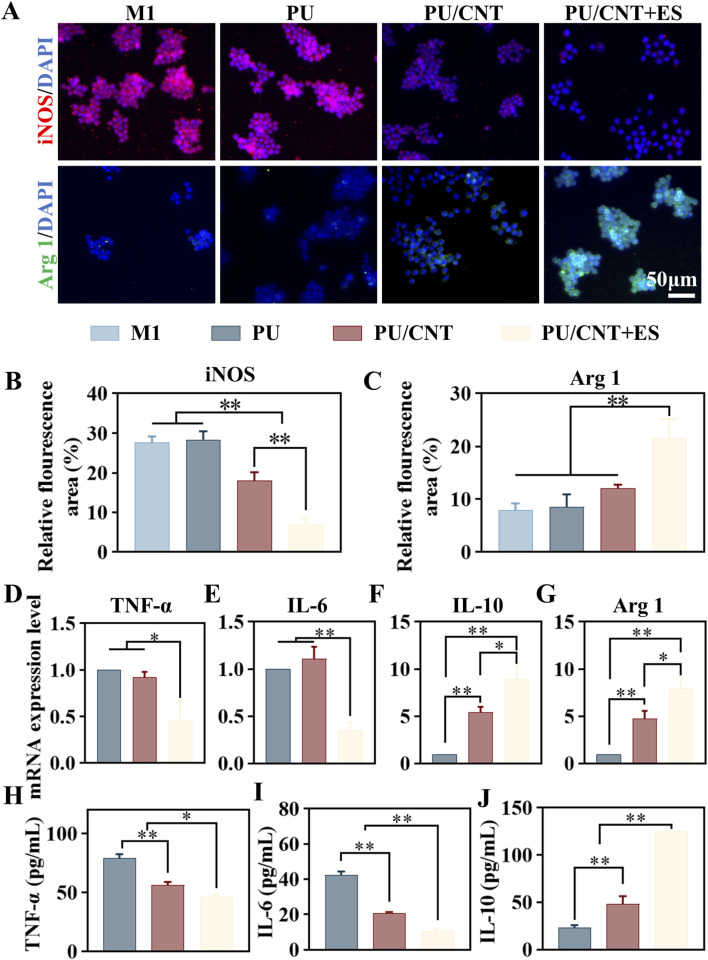
Impact of ES on macrophage phenotypic transformation and cytokine secretion profile. **(A)** Immunofluorescence staining images of RAW264.7 cells on day 3, with quantitative analysis showing **(B)** the percentage of iNOS-positive stained area and **(C)** the percentage of Arg1-positive stained area (n = 3). PCR results of RAW264.7 cells on day 3 (n = 3) for TNF-α **(D)**, IL-6 **(E)**, IL-10 **(F)**, and Arg 1 **(G)**. ELISA results of RAW264.7 cells on day 3 (n = 3) for TNF-α **(H)**, IL-6 **(I)**, and IL-10 **(J)**. Data are expressed as mean ± SD; *p < 0.05; **p < 0.01.

Immunofluorescence staining was used to detect the expression of iNOS and Arg1, two marker enzymes for the M1 and M2 phenotypes of macrophages, respectively (Figures 3A–C). PU/CNT + ES group exhibited the weakest fluorescence in iNOS staining and the strongest fluorescence in Arg1. Compared with other groups, PU/CNT + ES group showed a significant upregulation of Arg1, accompanied by a downregulation of iNOS. The differences were statistically significant, suggesting that ES may promote M2 polarization. The results of PCR demonstrated that, compared to PU group, the relative mRNA expression levels of TNF-α and IL-6 in PU/CNT + ES group were decreased by approximately 54% (*p < 0.05) and 65% (**p < 0.01), respectively, while those of IL-10 and Arg-1 were upregulated by 8.927 times (**p < 0.01) and 7.940 times (**p < 0.01), respectively. Significant differences were also observed between PU and PU/CNT groups in the expression of IL-10 and Arg-1 (**p < 0.01) (Figures 3D–G). Furthermore, the results of the ELISA for quantifying the secretion of related inflammatory factors demonstrated that, compared to PU and PU/CNT groups, PU/CNT + ES group showed significant downregulation of the levels of TNF-α and IL-6 (*p < 0.05 or **p < 0.01) and upregulation of IL-10 (**p < 0.01). Significant differences were also observed between the PU and PU/CNT groups (*p < 0.05 or **p < 0.01) (Figures 3H–J).

### 3.4 Evaluation of ES on promoting wound healing

**FIGURE 4:**
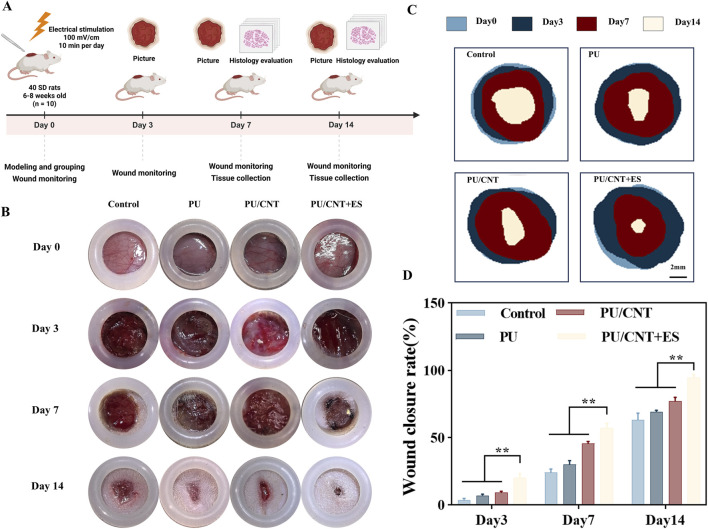
Impact of ES on promoting wound healing. **(A)** Schematic diagram of PU and PU/CNT nanofiber membranes combined with ES to promote wound healing. **(B)** Photographs of the healing process of skin wounds at 0, 3, 7, 14 days and **(C)** quantified maps of the healing process. **(D)** Healing process histogram at 3, 7, 14 days (n = 5). Data are expressed as mean ± SD; *p < 0.05; **p < 0.01.

After modeling and grouping, wound healing was observed and recorded on postoperative days 0, 3, 7, and 14 (Figure 4A). The results indicated that wound size decreased over time in all groups (Figures 4B,C). Compared with day 0, the wound healing rates in the Control, PU, and PU/CNT groups reached 63.85%, 69.55%, and 77.42%, respectively, by day 14. The PU/CNT + ES group exhibited a significantly higher wound healing rate (94.67%) than the other three groups (**p < 0.01) (Figure 4D). These findings demonstrate that PU/CNT + ES is more effective in promoting wound healing compared to PU and PU/CNT alone, indicating a positive role of ES in the wound healing process.

### 3.5 Histological analysis

**FIGURE 5:**
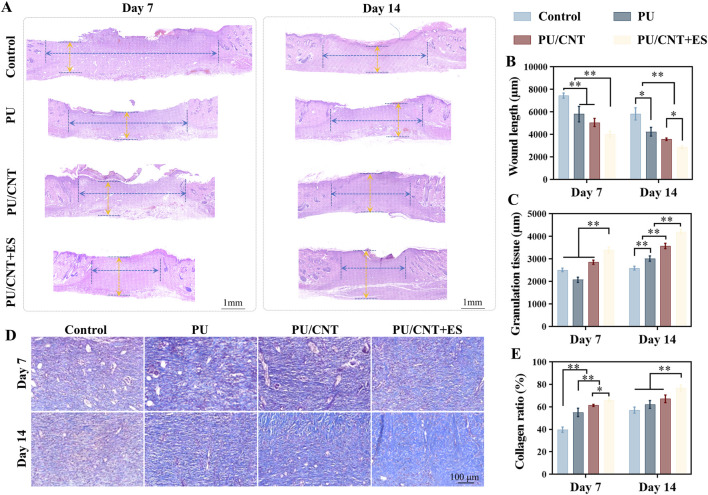
Histological analysis of Control, PU, PU/CNT and PU/CNT + ES group. **(A)** HE staining images of regenerated tissue sections from wounds at 7 and 14 days (Wound length, blue double-headed arrows; regenerated granulation tissue, yellow double-headed arrows) and the related **(B)** wound length and **(C)** granulation tissue thickness (n = 5). **(D)** Masson staining images of regenerated tissue sections from wounds at 7 and 14 days and the related **(E)** collagen ratio (n = 5). Data are expressed as mean ± SD; *p < 0.05; **p < 0.01.

As shown in Figure 5, compared with the other groups, the PU/CNT + ES group exhibited a narrower wound width, thicker granulation tissue, and a higher collagen ratio. As illustrated in Figure 5B, on day 14, the wound width in the PU/CNT + ES group (2870 ± 119.2 um) was significantly lower than that in control group (5840 ± 545.1 um), PU group (4249 ± 384.8 um), and PU/CNT group (3584 ± 110.0 um) (**p < 0.01). As depicted in Figure 5C, on day 14, the thickness of regenerated granulation tissue in the PU/CNT + ES group (4203 ± 122.6 um) was higher than that in control group (2597 ± 82.39 um), PU group (3025 ± 118.4 um), and PU/CNT group (3582 ± 119.7 um) (**p < 0.01). As presented in Figure 5E, on day 14, the collagen ratio in the PU/CNT + ES group (76.80% ± 2.775%) was higher than that in control group (57.20% ± 2.775%), PU group (62.20% ± 3.493%), and PU/CNT group (67.20% ± 3.421%) (**p < 0.01). These data indicate that the PU/CNT + ES group possesses a superior ability to promote wound healing compared to the other groups.

### 3.6 Immunohistochemical analysis

**FIGURE 6:**
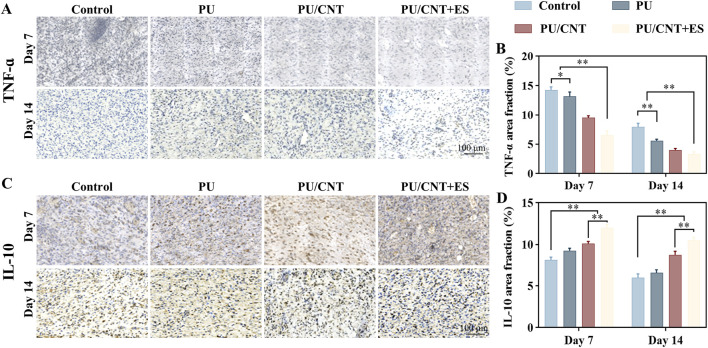
Immunohistochemical analysis of Control, PU, PU/CNT and PU/CNT + ES group. Immunohistochemical staining of **(A)** TNF-ɑ and **(C)** IL-10 at the wound site on day 7 and 14. **(B)** TNF-ɑ and **(D)** IL-10 immunohistochemical staining positive area. Data are expressed as mean ± SD; *p < 0.05; **p < 0.01.

Immunohistochemical analysis was used to further evaluate the effect of the nanofibrous scaffolds on promoting inflammatory regulation *in vivo*. As shown in Figures 6A–D, compared to Control, PU and PU/CNT groups, PU/CNT + ES group showed significant downregulation of the levels of TNF-α (**p < 0.01) and upregulation of IL-10 (**p < 0.01).

### 3.7 Evaluation of biosafety *in vivo*

**FIGURE 7:**
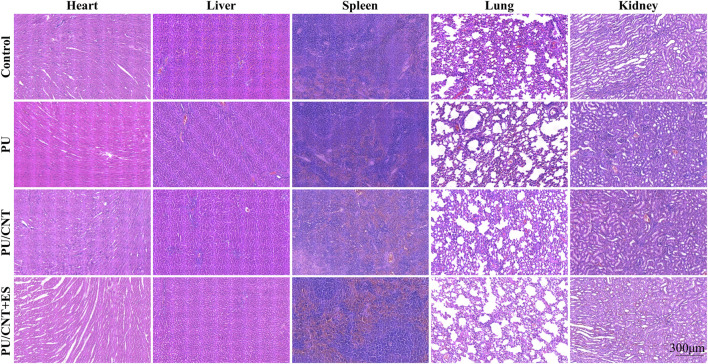
HE staining images of heart, liver, spleen, lung and kidney in Control, PU, PU/CNT, PU/CNT + ES group after 14 days of embedding.

In order to investigate the biotoxic effects of scaffolds on other organs, including heart, liver, spleen, lung and kidney, HE staining of those organs in Control, PU, PU/CNT and PU/CNT + ES group on day 14 was compared. HE staining results (Figure 7) showed that no obvious damage was found in the organs of rats with nanofibrous scaffolds embedded, indicating that nanofibrous scaffolds and its metabolites had no obvious adverse effects on the circulatory system, respiratory system, digestive system and metabolic system.

## 4 Discussion

Tissue repair is typically divided into four stages: clot formation, inflammation, new tissue formation (including re-epithelialization and granulation tissue formation), and tissue remodeling and resolution, with each stage involving multiple cell types (Peña and Martin, 2024). Macrophages, a heterogeneous population of myeloid cells, play numerous coordinating roles in the repair process. Currently, strategies for tissue repair primarily focus on the intrinsic properties of the materials themselves, including the natural mimicry of scaffolds, moisturizing properties, porosity, biodegradability, and biocompatibility (Hao et al., 2024), as well as the loading capabilities of the materials, including the ability to carry stem cells, drugs, and factors (Beavers et al., 2015; Yu et al., 2023). However, research on strategies involving additional physical stimuli remains inadequate. Nevertheless, it is worth noting that all cells in the body, not only excited nerve and muscle cells, can generate and receive steady-state bioelectric signals, which are key factors for regulating and controlling the quantity (cell proliferation and apoptosis), location (migration and orientation), and type (cell differentiation) of each cell (Liang et al., 2023). Therefore, exogenous ES may represent a new strategy to mimic the regenerative microenvironment in the body and promote tissue repair. In this study, we designed a tissue repair strategy combining a PU/CNT scaffold with excellent conductivity and exogenous ES and discovered its effective role in tissue repair.

This study successfully prepared PU/CNT nanofiber scaffolds using electrospinning technology. These scaffolds feature parallel-aligned fibers and well-organized surface structures, resembling the complex architecture of the extracellular matrix. The nanofiber scaffolds exhibit high porosity, providing ample space that is conducive to promoting cell proliferation and differentiation (Chen et al., 2016; Han et al., 2024). PU has excellent biomechanical properties, providing appropriate tensile strength, fatigue resistance and flexibility for tissue repair, and enabling better structural support compared to hydrogels (Nasab et al., 2022). Previous research has demonstrated that composite nanofiber scaffolds containing CNTs can spatially guide cell growth and enhance signal transmission, thereby facilitating tissue repair *in vitro* (Huang et al., 2020; Fabbro et al., 2013). In this study, CNTs were integrated to improve the conductivity and mechanical strength of the nanofiber scaffolds. The incorporation of CNTs significantly increased the electrical conductivity of the scaffolds from 10 to 6 S/m (PU group) to 100 S/m (PU/CNT group), providing an ideal platform for effective ES delivery. Additionally, SEM and TEM results revealed that both groups of fiber scaffolds exhibited good orientation, indicating that the addition of CNTs did not affect the orientation of the scaffolds. The orientation of nanofiber scaffolds serves as an important topographical guidance cue that plays a crucial role in processes such as cell directional growth and migration (Jin et al., 2023; Huang L. et al., 2018; Zhao et al., 2024). At the same time, the highly oriented structure can optimize the conduction and output performance of ES on the scaffolds (Zhang M. et al., 2023). Overall, PU/CNT scaffolds demonstrated the expected electrical conductivity and *in vivo* microenvironment simulation, creating a favorable microenvironment for tissue repair.

In the early stages, tissue macrophages produce mitochondrial ROS and drive several early repair processes, amplifying the inflammatory response. Later, the characteristics of macrophages are mitochondrial respiration and catabolic functions, suppressing inflammation, regulating ECM deposition, and promoting tissue repair (Willenborg et al., 2021; Sheppe and Edelmann, 2021). Excessive early inflammation can lead to excessive ROS production after injury, which disrupts calcium homeostasis, causes lipid peroxidation of cell membranes, and leads to damage to nucleic acids and proteins (Xia et al., 2021). In addition, ROS can promote M1 polarization of macrophages, which in turn activates inflammatory activation through multiple signaling pathways such as STAT 1, NFκB, p38 MAPK, and NLRP 3 inflammasome (Pawate et al., 2004; Padgett et al., 2014). Evidently, regulating the inflammatory response and promoting the anti-inflammatory transition of macrophages is crucial in tissue repair, and the dynamic transition in macrophage phenotype from pro-inflammatory to anti-inflammatory plays a key role (Peña and Martin, 2024). When macrophages polarize, they differentiate into functionally opposite M1 and M2 macrophages. M2 macrophages express increased levels of Arg-1, mannose receptor (CD206), IL-10, and chemokines CCL17 and CCL22, which play significant roles in tissue repair, angiogenesis, and metabolism (Yunna et al., 2020). Therefore, the combined strategy designed in this study aims to alleviate the harmful effects of excessive inflammation and promote macrophage M2 polarization to facilitate the tissue repair process.

In the selection of macrophage models for experimental design, various macrophage lines exhibit distinct advantages and disadvantages. Among them, RAW264.7 and THP-1 are widely utilized macrophage models (Chen et al., 2017; Liu X. et al., 2021; Fan Q. et al., 2022). THP-1 is a human myeloid leukemia monocytic cell line, and RAW264.7 is a mouse leukemia monocytic cell line derived from myeloid cells (Chen et al., 2023a). RAW264.7 cells, originating from mouse leukemic monocytes, demonstrate high sensitivity to physical/chemical stimuli such as ES and nanomaterials (Chen et al., 2023a). However, polarization macrophages still have significant differences between human and mouse cell lines (Li P. et al., 2021). The advantage of human THP-1 cells lies in being closer to the human pathological physiological environment, but they need to be induced to differentiate into macrophage-like states by PMA, which may introduce additional variables and have lower phenotypic stability (Li P. et al., 2021; Chanput et al., 2014). Therefore, if focusing on clinical translation, THP-1 cells should be used to verify key mechanisms to compensate for the differences in human and mouse immune responses. If the focus is on high-throughput screening or mechanism exploration, RAW264.7 is still the preferred choice due to its operational simplicity (Li P. et al., 2021). In this study, we utilized RAW264.7 cells as the cellular model for initial mechanistic exploration. In the future, a multi-model complementary strategy, such as initial screening with mouse-derived cells followed by validation with human-derived cells, will further enhance the reliability and generalizability of research.

Good biocompatibility is the foundation for the function of biomaterials. It refers to the appropriate host response during the expected clinical usage period in the body. In tissue engineering, scaffolds provide support for cell attachment, proliferation, and differentiation. Therefore, if cells adhere to and migrate across the surface and through the extracellular matrix of the scaffold, followed by cell proliferation and deposition of new matrix, the tissue engineering scaffolds and implant materials can be considered biocompatible (Munir et al., 2019). In this study, WMCNTs were utilized to impart conductive properties to PU nanofibrous scaffolds due to their unique mechanical, physical, and electronic characteristics. However, the safety and toxicity of WMCNTs remain controversial. Some studies have reported the formation of granulomas in the lungs of mice administered with different concentrations of CNTs, exhibiting lung-related cytotoxicity (Lam et al., 2004; Warheit et al., 2004). Conversely, other studies indicate that CNTs can purposefully interact with proteins, nucleic acids, and cells, demonstrating good cytocompatibility and playing a positive role in tissue repair (Munir et al., 2019). CCK-8 and Live/Dead staining are commonly used reagents to evaluate scaffold cell proliferation and cytotoxicity (Yi et al., 2024; Ja et al., 2022). The results of this study show that cells in all groups exhibited good proliferation and survival rates. The addition of CNTs did not affect the values of CCK-8 and even promoted cell proliferation. This could be attributed to various factors influencing the toxicity level of CNTs in the physiological environment, including metal catalysts and processing methods (Li et al., 2013). After integration into PU scaffold via electrostatic spinning technology, CNTs appeared non-toxic under physiological conditions. Additionally, their conductivity and ES exhibited a synergistic effect, thereby promoting cell proliferation. In the evaluation of *in vivo* biocompatibility, H&E staining of organs in each group of rats revealed no significant damage, further confirming the biosafety of the nanofiber membrane.

Numerous studies have demonstrated the beneficial effects of ES in tissue repair (Hoare et al., 2016; Li J. et al., 2021). iNOS and Arg1 serve as markers for M1 and M2 macrophages, respectively (Kieler et al., 2021). In this study, immunofluorescence experiments revealed that ES can upregulate Arg1 expression while downregulating iNOS expression, indicating that ES promotes M2 polarization of macrophages, which is consistent with previous findings that ES can modulate macrophage phenotype by inhibiting the MAPK/JNK signaling pathway and activating oxidative phosphorylation and ATP synthesis (Wu et al., 2023). Besides, observations of wound healing in animal experiments, along with results from HE and Masson staining, demonstrated that ES can reduce wound length, regenerate granulation tissue, and increase collagen ratio, thereby facilitating wound healing. The adjustment of ES parameters is crucial for exerting these effects. Therefore, setting the safest and most effective ES parameters, such as voltage, frequency, time, ES mode, is a key point for future research. Low voltage (<50 mV/mm) has limited effectiveness, while excessively high voltage (>200 mV/mm) may damage cells (Zhao et al., 2020). ES at different frequencies also exhibits varying effects. You et al. found that the cell survival rate was highest in the 500 Hz ES group (You et al., 2019). Additionally, different ES modes, including direct current (DC), alternating current (AC), pulsed current (PC), and pulsed electromagnetic fields (PEMF), have been proven to have beneficial effects in tissue repair (Thakral et al., 2013; Zhao et al., 2006; Zhao, 2009), but their impact on macrophage metabolism and phenotypic transformation remains unclear.

In mechanistic studies, PU/CNT nanofiber scaffolds upregulated the expression of genes associated with the M2 phenotype of macrophages (such as Arg1 and IL-10 (Opal and DePalo, 2000)) and suppressed the expression of genes linked to the M1 phenotype (such as TNF-α and IL-6 (Liu C. et al., 2021)) through the transmission of exogenous ES signals. ELISA is an immunoassay technique for the quantitative detection of antigens or antibodies in biological samples (Lequin, 2005), and its results also provides compelling evidence that exogenous ES signals can enhance the expression of the anti-inflammatory protein IL-10 and reduce the expression of the inflammatory proteins TNF-α and IL-6. *In vivo* immunohistochemical analysis further confirmed ES promoted the expression of IL-10 and suppressed the expression of TNF-α. These findings suggest that ES promotes M2 polarization of macrophages, further modulating their cytokine secretion profile and shifting macrophage function from pro-inflammatory to anti-inflammatory. On the other hand, IL-10 is commonly used to induce M2 polarization of macrophages *in vitro* (Huang X. et al., 2018). Numerous studies have also shown that the IL-10 factor is a key factor regulating the activation pathways of macrophages, and its high expression has been confirmed to promote M2 polarization of macrophages *in vivo* and *in vitro* (Chen et al., 2023b; Li et al., 2022; Sun et al., 2024). This indicates that macrophage polarization and cytokine secretion by macrophages have a mutually reinforcing effect, jointly reshaping the immune microenvironment during regeneration.

The coherent alignment between our *in vitro* and *in vivo* findings strongly suggests that the promoted wound healing phenotype observed in rats is mechanistically rooted in the ES-driven immunomodulation at the cellular level. While our data clearly demonstrate that the PU/CNT + ES group promotes an M2-like cytokine profile, an intriguing question remains regarding how the physical cue of ES is transduced into a sustained pro-regenerative macrophage phenotype. Beyond the potential signaling pathways like MAPK/JNK (Wu et al., 2023), recent insights into immunometabolism propose that the functional polarization of macrophages is underpinned by a fundamental metabolic reprogramming (Bianconi et al., 2024; Srirussamee et al., 2019). Typically, M1 macrophages exhibit enhanced glycolysis, while M2 cells show increased fatty acid oxidation and oxidative phosphorylation (Viola et al., 2019). It is plausible that ES not only provides topographical and electrical cues but may also orchestrate a shift in macrophage metabolic state. For instance, ES may promote a metabolic shift from glycolysis towards oxidative phosphorylation, a shift characterized by the M2 phenotype (Bianconi et al., 2024). This metabolic reprogramming could improve the stability of the anti-inflammatory state and the consequent secretion of pro-regenerative factors like IL-10, thereby directly linking the observed *in vitro* polarization to the enhanced tissue repair *in vivo*. Future studies focusing on the metabolic landscape of macrophages under ES will be crucial to unravel this intricate connection.

In summary, the PU/CNT electrospun conductive nanofiber scaffold combined with exogenous ES exerts an inhibitory effect on inflammatory responses and accelerates tissue repair by regulating macrophage phenotype polarization and cytokine secretion profiles. This provides a theoretical foundation and practical guidance for the development of novel immunomodulatory nanofiber scaffolds. In the future, it is necessary to further optimize the parameters and types of ES and explore more complex models, such as the efficacy of the scaffold in diabetic wounds, promoting the clinical translation of nanofiber scaffolds.

## 5 Conclusion

In this study, conductive PU/CNT nanofiber scaffolds were successfully fabricated by electrospinning technology. Combined with exogenous ES (150 mV/mm), it was confirmed that these scaffolds could significantly promote the polarization of macrophages to the M2 phenotype, manifested by the upregulation of Arg1 expression and the downregulation of iNOS, while the secretion of pro-inflammatory factors (TNF-α, IL-6) was reduced and the anti-inflammatory factor (IL-10) was increased. *In vivo* studies further demonstrated both the biological safety of the scaffolds and its efficacy in promoting immune regulation and wound healing. This finding provides experimental evidence for the development of novel immunomodulatory biological scaffolds. Future research can focus on optimizing ES parameters, such as frequency and mode, and incorporating growth factor loading strategies to further advance their clinical application in tissue repair.

## Data Availability

The original contributions presented in the study are included in the article/supplementary material, further inquiries can be directed to the corresponding authors.
